# How to Become a Mentalist: Reading Decisions from a Competitor’s Pupil Can Be Achieved without Training but Requires Instruction

**DOI:** 10.1371/journal.pone.0073302

**Published:** 2013-08-26

**Authors:** Marnix Naber, Josef Stoll, Wolfgang Einhäuser, Olivia Carter

**Affiliations:** 1 Neurophysics, Philipps-University, Marburg, Germany; 2 School of Psychological Sciences, University of Melbourne, Parkville, Victoria, Australia; 3 Vision Sciences Laboratory, Harvard University, Cambridge, Massachusetts, United States of America; 4 Cognitive Psychology Unit, Leiden University, Leiden, The Netherlands; 5 Center for Interdisciplinary Research (ZiF), Bielefeld, Germany; University of Turin and the Italian Institute of Technology, Italy

## Abstract

Pupil dilation is implicated as a marker of decision-making as well as of cognitive and emotional processes. Here we tested whether individuals can exploit another’s pupil to their advantage. We first recorded the eyes of 3 "opponents", while they were playing a modified version of the "rock-paper-scissors" childhood game. The recorded videos served as stimuli to a second set of participants. These "players" played rock-paper-scissors against the pre-recorded opponents in a variety of conditions. When players just observed the opponents’ eyes without specific instruction their probability of winning was at chance. When informed that the time of maximum pupil dilation was indicative of the opponents’ choice, however, players raised their winning probability significantly above chance. When just watching the reconstructed area of the pupil against a gray background, players achieved similar performance, showing that players indeed exploited the pupil, rather than other facial cues. Since maximum pupil dilation was correct about the opponents’ decision only in 60% of trials (chance 33%), we finally tested whether increasing this validity to 100% would allow spontaneous learning. Indeed, when players were given no information, but the pupil was informative about the opponent’s response in all trials, players performed significantly above chance on average and half (5/10) reached significance at an individual level. Together these results suggest that people can in principle use the pupil to detect cognitive decisions in another individual, but that most people have neither explicit knowledge of the pupil’s utility nor have they learnt to use it despite a lifetime of exposure.

## Introduction

The notion of “mind-reading” has long been a theme within popular culture but with the development of new brain imaging methods for “decoding” what a person is seeing or thinking (e.g., [1,2]), "mind-reading" has begun to move into the scientific mainstream. The success of these methods has also renewed interest in the question to what extent subtle facial signals may provide clues into one’s private thoughts. It is well-established that facial expressions and gaze direction can reveal some information about an individual’s emotional state or intention [3,4]. One question that remains open, however, is whether pupil dilation can also be used to gain strategic insights into another’s mind.

It has been shown that observers tend to mirror their pupil size to those of others [5] suggesting that humans have an autonomic system that is responsible for implicit (subconscious) monitoring of others’ pupil sizes [6,7]. These systems may further play a specific role during the unconscious processing of socially relevant cues such as emotions [5,8] and attractiveness [9–15]. These studies thus imply that the pupil is a potential source of information during interpersonal interactions, at least in an emotional context. However, it remains unknown whether observers can exploit pupil dynamics in other, non-emotional circumstances.

Pupil dilation is known to accompany a wide range of behaviors and mental processes, including load [16], arousal [17], alertness [18], working memory load [19–21], attention [22,23], familiarity [24], emotions [25–28], high-level visual processing [29], and making a conscious decision [30–34]. It has also been demonstrated recently that eye tracking cameras can capture small changes in pupil size that predict cognitive events such as an act of deception [35] and the timing of decisions [33]. Here we examine whether human observers can - and do - extract similar information about another individual’s cognitive decisions from these subtle changes in pupil diameter.

**Figure 1:**
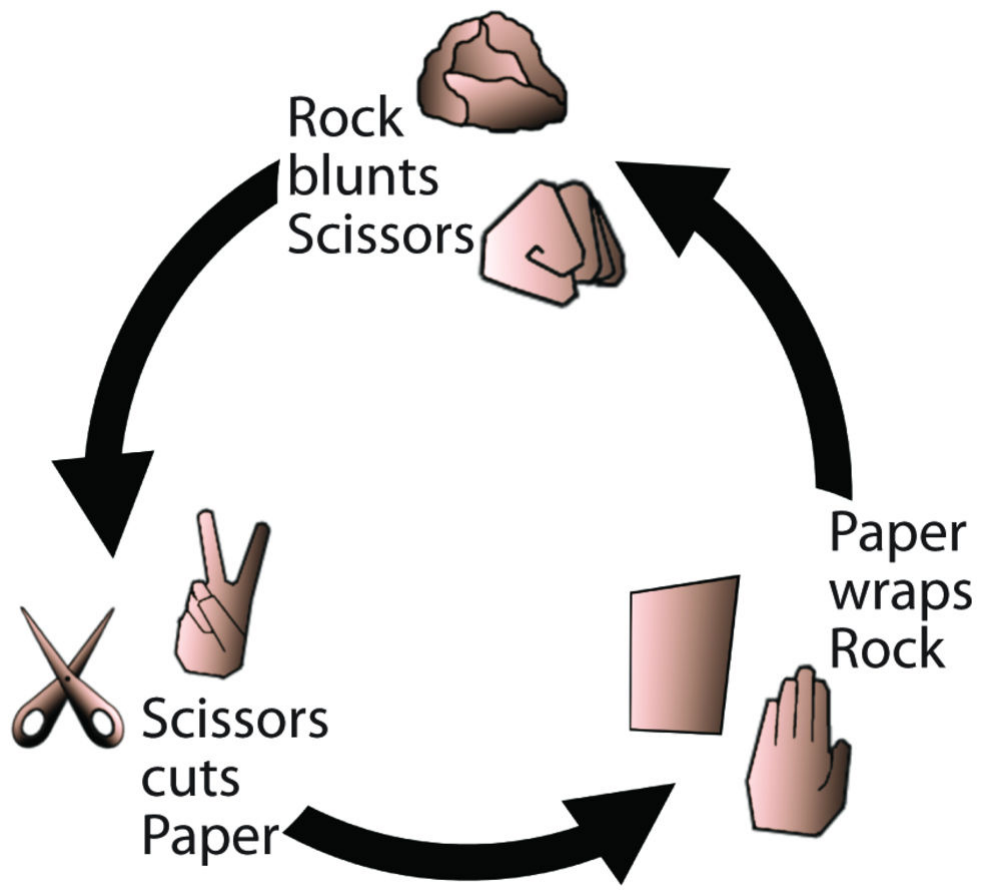
Schematic illustration of the rock-paper-scissors game. In a game of rock-paper-scissors, the relative choices of the two competitors determine the outcome.

To test this we modified the popular childhood game Rock-Paper-Scissors (RPS) (see Figure 1). In the original version of the game participants are required to make their decisions simultaneously. One feature of the traditional version is that it is possible to gain an advantage by taking past decisions into account because people have difficulty generating random response sequences in general [36] and in the context of RPS [37]. To ensure that the prior history of events was not informative in the context of this experiment, we pre-recorded a sequence of 75 games played by 3 “opponents” (25 games each) and played them back to a new set of players in a random order. Critically, the response of the pre-recorded opponent remained concealed during the game and was revealed immediately after the player had indicated their own choice of Rock, Paper or Scissors. Therefore, while the temporal sequence of the game was radically altered, this modification allowed us to maintain the critical elements of the game (concealed mutual decision in a competitive game environment), while randomizing the order of game presentation to ensure that the prior sequence of decisions was uninformative.

Using this modified version of RPS, here we demonstrate for the first time that people can use the pupil to detect cognitive decisions in another individual, but that most people have to first be made explicitly aware of the strategic information provided by pupil dilation.

## Materials and Methods

### 2.1: Ethics Statement

The experiments were approved by the ethics committees of University of Melbourne and Philipps-University, Marburg’s department of psychology, and conformed to the ethical principles of the Declaration of Helsinki.

### 2.2: Participants

**Figure 2:**
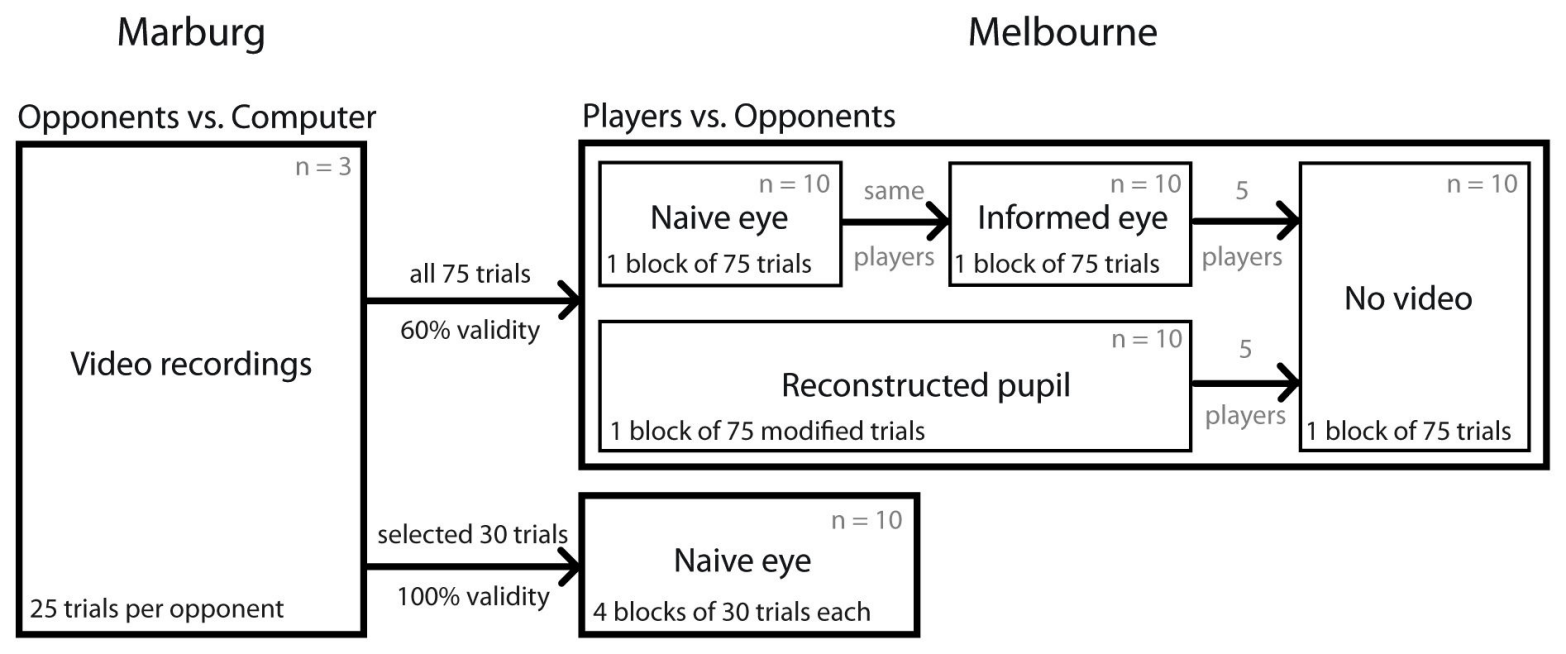
Participant breakdown. Three opponents each played 25 modified RPS games against a computer. During these games video of the opponents’ eyes was recorded along with the audio produced by the computer. For all conditions, except “100% validity”, all 75 of these games were used. For the 100% validity condition, 30 (out of 45) videos in which maximum pupil dilation indeed followed the choice of the opponent were selected with 10 games for each of the 3 intervals respectively. These valid games were then randomly presented as in the original "naïve eye" condition. Ten players participated first in the "naïve eye" and subsequently in the "informed eye" condition to allow a within-subject comparison. A distinct set of 10 players participated in the reconstructed pupil condition. Half (5) of each group participated in addition in the "no video" control. Finally, a distinct set of 10 players participated in the 100% validity condition.

In total 33 volunteers (age: 19-30) participated in the study. Three of them served as "opponents" in that their responses were used to generate the stimuli used in the main experiments. These opponents were recruited from the Philipps-University Marburg, where the filming was conducted and the stimuli were generated. The remaining 30, filling the role of "players", were recruited and tested in Melbourne, Australia. The 30 players each participated in one or more of the experimental conditions as indicated in Figure 2. All participants were naïve to the purpose of the experiments, gave written-informed consent before participation, and received payment for participation in addition to performance-dependent reward ($10-$20).

### 2.3: Procedure

#### 2.3.1: Rock-paper-scissors rules

The two-player game had a straightforward rule structure, which all participants understood with no training. The outcome (win/loss/draw) of each game was determined by the relative choices. Rock wins against scissors, scissors against paper, paper against rock (Figure 1). If both competitors chose the same option, the game was drawn.

#### 2.3.2: Stimulus construction (Opponents’ games)

In a first phase, we constructed stimuli by recording videos of the pupil dilations of 3 participants ("opponents") who each played 25 games of rock-paper-scissors. The opponents played against a computer in a room with low ambient light levels. The words "rock", "paper", "scissors" were read out by a text-to-speech-voice converter and presented through computer speakers at comfortable listening volume. Opponents were presented the audio track of "rock", "paper", "scissors" in random order with 4-s intervals between each word onset, and were asked to indicate their choice by pressing a button immediately after the respective audio word was presented. At the completion of each game (i.e., 4s after the last option was read out), the computer’s (random) choice was shown on the screen and feedback about the resulting outcome (win/loss/draw), and the associated monetary reward was provided. Throughout the rest of the game the screen remained a blank uniform grey. During each game, video of their left eye was recorded by a Grasshopper GRAS-03K2M camera (Point Grey Research, Richmond, BC, Canada) at 120 Hz and 640x480 resolution and stored together with the presented audio track (Movie S1). It was these recorded movies of the opponents left eye that served as stimulus material for the main experiments.

**Figure 3:**
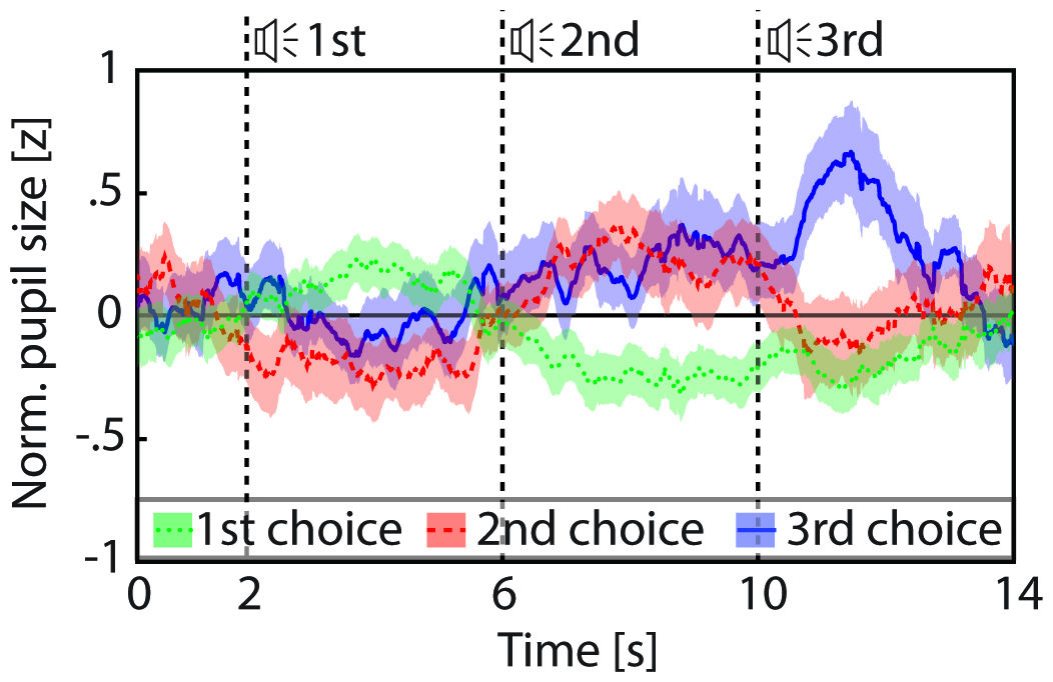
Pupil responses to cognitive decisions. Mean pupil size (diameter, normalized to z-scores within each opponent) and its standard error (shading) for games in which opponents selected the first, second or third option; average pupil dilation peaks shortly after the presentation of the selected word (dashed vertical lines).

**Figure 4:**
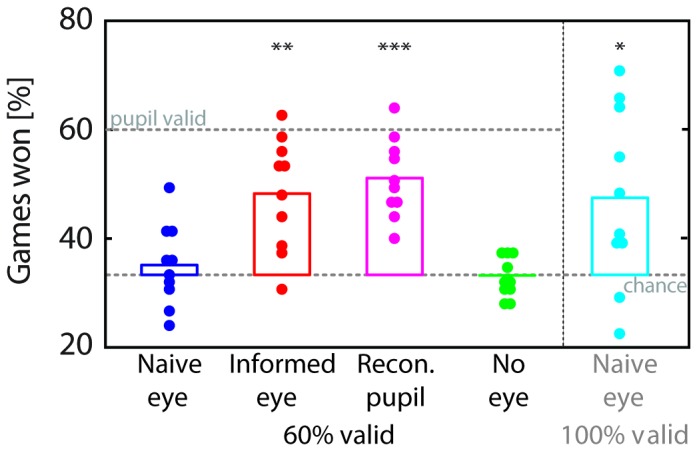
Group performance across conditions. Percentage of games won on average (bar) and by individual players (circles). For the 60%-validity conditions upper dashed line indicates maximum performance if a player always selected the interval signaled by the pupil (correct in 45/75 games).

To verify whether the time of maximal pupil dilation was indeed informative about an opponent’s choice, we first analyzed the opponent’s responses and their corresponding pupil dilation. Opponent’s choices were spread approximately evenly over the 3 intervals with 28, 25 and 22 selections for first, second and third option, respectively. Analysis showed that the opponents’ pupil size varied substantially throughout their games despite minimal variability in external light sources (opponent’s viewed a blank screen in a dimly lit room during all games). On average the pupil measured 4.6mm during the games, and the difference between minimum and maximum in each game amounted to 3.8mm on average (SD across games: 1.0mm). In 45 out of 75 games, maximum pupil dilation followed the selected word (Figure 3). Hence, the time of maximum dilation was a significant marker for the opponent’s choice with a validity of 60% (45/75 games, compared to chance level of 25/75 – indicated by the dashed lines in Figure 4), which is compatible with earlier data obtained in a free choice scenario [33].

#### 2.3.3: Conditions (Players’ games)

A separate group of 30 observers ("players") - distinct from the "opponents" - played rock-paper-scissors against the video recordings of the opponent’s games under a number of conditions. Players sat in a room with low ambient light levels at a distance of about 120cm from the screen with their head stabilized by a chin rest. Videos (8-bit grayscale, 640x480pixels@60Hz on a 1920x1200 pixel, 52x32.5cm wide TFT screen) were presented centrally subtending a visual angle of about 4° x3°, with the pupil covering about 0.25° to mimic real-life conversation distance of about 50cm [38]. In all conditions, each game consisted of the same audio track ("rock", "paper", "scissors" in random order with 4-s intervals) that had been presented to the respective opponent during the corresponding game.

Players were instructed to report their choice ("rock", "paper" or "scissors") via button response at the end of each game (i.e., approximately 4s after the last option had been read out). Immediately after the players’ choice had been indicated, their opponent’s selection was shown on the screen and feedback about the resulting outcome (win/loss/draw), and the associated monetary reward was provided. Importantly, as the opponent’s choices were recorded during the video recording phase, the feedback provided to players was accurate with respect to the original opponent’s games.

In all conditions, the games were presented in random order such that no information was provided by the previous pattern of choices, and players were correctly informed about this fact prior to the experiment. The visual information available to the player depended on condition:

- (1) *Naïve-eye* condition: To assess whether naïve participants had any explicit or implicit knowledge about the utility of the pupil, players were shown the full videos of their opponents’ left eye and were instructed simply to "look for any behavioral signs that could reveal the opponent’s decision."
- (2) *Informed-eye* condition: To determine whether pupil dilation could, in principle, be used to gain a strategic advantage, players were shown the full videos of their opponents’ left eye and were informed that “the largest pupil dilation should follow the presentation of the word selected by the recorded opponent".
- (3) *Reconstructed pupil* condition: The video was replaced by a black disk on a gray background that matched the pupil size at each point in time (Movie S2). As this condition was otherwise identical to the "informed-eye" condition, it served as an important control to rule out any contribution of non-pupil factors (such as blinks or facial movements) in the players’ performance.
- (4) *No-video* condition: Players were also presented the same audio track of the RPS games while viewing a blank grey screen (in all other respects the basic procedure was the same). Consistent with the previous conditions the players were asked to indicate their selection immediately after the presentation of the audio track and they were provided correct feedback after each game. This condition served as control to test that indeed no information was available from the audio sequence or any biases/patterns in the opponent’s choices.
- (5) *Naïve-eye* (*100% validity*) condition: This condition aimed to determine whether observers could ever learn to use the signal, by using the most optimal conditions. This served as a control to show that there was no technical or perceptual limitation that prevented players from using the pupil in the naïve-eye 60% validity condition. Unlike in the four aforementioned conditions, which used all the games recorded from the opponents (i.e., irrespective of whether the pupil correctly signaled the option chosen by the opponent), here 30 games were selected. These were selected by first identifying the 45 “valid” games - for which maximum pupil dilation followed the selected option. From these 45 games we selected the first 10 games in chronological order that corresponded to each of the first, second or third interval. Instructions were identical to the original naïve-eye condition. This condition allowed us to provide optimal feedback to test whether the players could ever learn to exploit the pupil signal in our RPS game scenario. Since only 30 trials were available, these were repeated four times in separate blocks and randomized independently within each block.

### 2.4: Data analysis

Average performance in each condition was compared against chance (33%) by a two-sided t-test. Average performance between original *naïve-eye* condition and *informed-eye* condition (same set of players) was compared by a paired two-sided t-test, other comparisons between conditions (partially or fully distinct set of players – see Figure 2 for participant breakdown) by unpaired two-sided t-tests. The unpaired tests, which for the comparison to the "no eye" condition are less powerful, and thus more conservative with respect to the hypotheses considered.

To test whether individuals performed significantly above chance in a condition, irrespective of whether the group on average succeeded in doing so, we compared individual performance over the course of each experiment against chance by means of a Binomial test. A Binomial test is an exact statistical test that provides the probability (p) that for a certain total number of games(n), a certain amount of wins (k) or more are to be expected by chance. For the present case of 1/3 chance, the (one-sided, as learning can be expected to improve performance) p-value is thereby given to $p=\sum_{k\leq i\leq n}\left(\begin{array}{c} n\\ i \end{array}\right){\left(\frac{1}{3}\right)}^{i}{\left(\frac{2}{3}\right)}^{\left(n-i\right)}$. Since 10 tests have to be performed in each condition, we refer to a result as "significant", only if the p-value is below 0.005, corresponding to a Bonferroni-corrected alpha-level of 5%.

## Results

### 3.1: Players can readily exploit opponent’s pupil signal, but most require instruction

#### 3.1.1: Average performance

Ten players competed against the original unedited movies of the opponents’ games, including the original "rock"-"paper"-"scissors" audio track and the corresponding video of their left eye (Movie S1). When no instruction was given regarding the pupil (naïve-eye condition), their average performance was indistinguishable from chance (Figure 4, dark blue; *t*(9) = 0.73, *p* = 0.48). When the same 10 players repeated the experiment with instruction to look for the maximum pupil dilation ("informed-pupil"), their performance improved to significantly better than both chance (Figure 4, red; *t*(9) = 4.56, *p* = 0.001) and performance in the naïve-eye condition (t(9) = 3.03; *p* = 0.01).

A group of 10 new players also performed clearly above chance when they were presented movies of the “reconstructed pupil” represented by an expanding/constricting black disk on gray a background (Movie S2), (Figure 4, magenta; *t*(9) = 7.71, *p* = 3.02x10^-5^). This condition merely served to test whether participants were not exploiting any potentially useful signals other than the pupil. As observers performed as well as in the “instructed-pupil” condition in the absence of all other cues, this suggests that the pupil is at least as informative as in combination with other facial information.

To ensure that none of the players could exploit any possible sequential order of the opponent’s choices, in all conditions the order of games had been randomized. To further ensure that there was no possible strategy involved, we tested 5 players of each player group in an additional control condition ("no eye"), in which only the audio track was presented for the identical collection of games tested in the previous conditions. In the absence of any visual information, performance was indistinguishable from chance (*t*(9) = 0.46, *p* = 0.66, Figure 4, green) and significantly worse than in the "informed-eye" (*t*(18) = 4.45, *p* = 3.10x10^-4^) and the "reconstructed-pupil" (*t*(18) = 7.09, *p* = 1.32x10^-6^) conditions, but indistinguishable from the naïve-eye condition (*t*(18) = 0.86, *p* = 0.40). This control verified that there was indeed no information beyond the video signal that was being exploited by players.

#### 3.1.2: Individual performance

**Figure 5:**
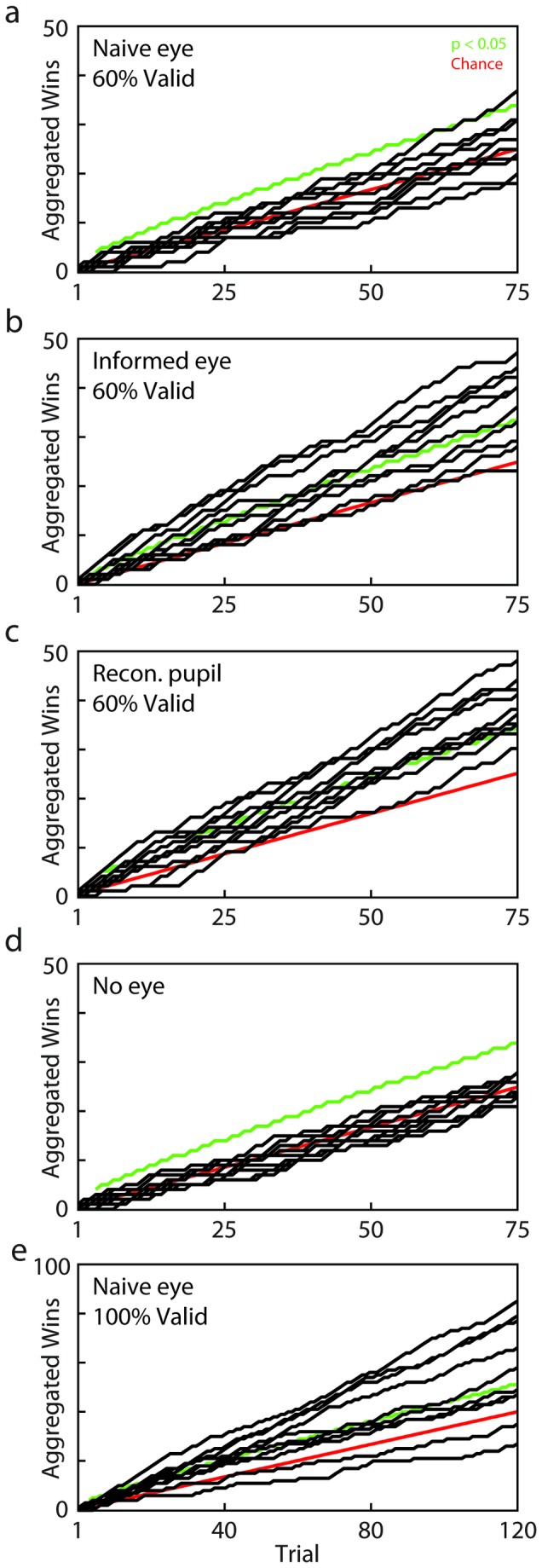
Individual performance across conditions. Learning curves for the 5 different experimental conditions (*Black lines*: aggregate number of wins for each individual; *red line*: chance level; *green line*: 5% significance level for Binomial test at the given number of games). Note that all preceding games are considered in this computation, such that learned information has to persist longer if learning starts later. **a**) naïve-eye **b**) informed-eye **c**) reconstructed-pupil d) no-eye control condition **e**) naïve-eye (100% validity).

While there was no indication that the average *naïve-eye* player could exploit pupil dilation, the question remained whether a "lucky few" individuals could learn to use the pupil without explicit instruction. Testing individual performance over the course of the naïve-eye experiment by means of a Binomial test, we found one individual in the naïve-eye condition that indeed increased their chance of winning significantly above chance (37/75 wins, p=0.003, Figure 5a). In comparison, by the end of the 75 games, 7/10 players reached significance at an individual level for "informed-eye" (all 7p<0.0005, Figure 5b) and 9/10 for reconstructed-pupil (all 9p<0.0005, Figure 5c). In the control "no-eye" condition, no individual showed any sign of learning (Figure 5d, all p>0.05).

### 3.2: More players learn to exploit the pupil without instruction, if its validity is increased

While the timing of maximum dilation, which observers were instructed to look for, was informative about an opponent’s choice in the majority of games, such 60%-validity is far from perfect. Most players reported in debriefing after the naïve-eye condition that they had tried using pupil-related cues, but varied strategies between looking for constrictions and dilations, and intermixed information from other facial signals, events like blinks, eye-brow twitches, and head movements. This raised the question as to whether the lack of learning in most individuals was a consequence of the relative proportion of valid and invalid games. To test whether players could theoretically ever spontaneously learn to use the information conveyed by the pupil, we selected 30 games (10 from each response interval) for which maximum pupil accompanied correct choice (i.e., 100% validity). A fresh set of 10 naïve players played 4 randomized blocks of these 30 games. In all other respects this "100%-validity" naïve-eye condition was identical to the original naïve-eye condition. Unlike in the original 60% validity naïve-eye condition, players significantly performed above chance on average (Figure 4, cyan; t(9) = 2.77, *p* = 0.022) and better than in the *no-video* condition (*t*(18) = 5.22, *p* = 5.77x10^-5^). On an individual level, 5 out of 10 individuals significantly performed above chance, showing clear signs of learning (Figure 5e; p < 0.0005). This shows that, in the absence of any instruction, people can – in principle – spontaneously learn to use information signaled by another individual’s pupil. Nonetheless, as such high validity is unlikely to occur in real-world situations this condition serves as a control to verify that there is no principled inability to extract useful information for learning, when the pupil is embedded in its natural context. Indeed, the fact that the players show no clear ability to use this signal in the naïve condition, despite a life-time of exposure to other people’s pupils, suggests that it is extremely unlikely that they are currently utilizing this signal in daily life.

## Discussion

In the present paper we used an adapted version of the childhood game of rock-paper-scissors to demonstrate four key results. i) We filmed the left eye of a group of opponents as they played against a computer and confirmed earlier findings that pupil dilation increases at the time of a conscious choice. ii) We show, for the first time, that competitors can exploit the signal conveyed by another individual’s pupil. iii) As performance was equivalent for games in which the players viewed the unedited footage of their opponent’s eye or a reconstructed movie showing only the pupil diameter, it can be concluded that the information in the pupil must be at least as informative as that conveyed by the pupil plus other facial features. iv) It was found that – in principle – the information conveyed by the pupil can be learned without any explicit instruction. However, such learning requires conditions that are so constrained that a general implicit knowledge and use of this signal in daily life appears unlikely.

Pupil dilation in the context of a decision has been known for over half a century [39]. As we confirm here, the link between dilation and decision is timed with sufficient precision to convey a covert decision [33], suggesting that careful monitoring of the pupil may assist the detection of deceptive acts [35]. Evidence also shows that the observation of another individual’s pupil can influence the perception of emotions, such as sadness [5,8]. Such observed pupil dilation is known to modulate neural activity related to emotion processing, presumably without explicit awareness [5–8]. Despite this demonstrated link between pupil response and decision-making and the use of pupil dilation in an emotional context, no study has yet addressed whether competitors can *explicitly* exploit an opponent’s pupil dilation in a competitive scenario. Our results on the explicit use of pupil dilation are two-fold. On the one hand, we find that explicit usage of the pupil signal is clearly possible. Furthermore – as shown by the "reconstructed pupil" condition – the pupil alone is at least as informative as its combination with other facial features. On the other hand, however, nearly all naïve observers require explicit instruction to exploit the information the pupil conveys.

It is possible that the constrained nature of the experimental design has limited the relevance of this study to more realistic scenarios. The use of prerecorded rather than live opponents and the presentation being limited to the eye region rather than full faces might have created an unusual situation that failed to engage an implicit ability to link pupil dilations to decision-making. Although all of these factors need to be considered in future studies on the role of pupil dynamics in interactive contexts, we consider it unlikely that the procedural constraints undermined people’s awareness of the pupil’s cognitive signals in this instance. Firstly, players were able to instantly use the pupil once they were made aware of the association between dilations and decision-making. This shows that their inability to pick up the pupil cue prior to instruction – despite exposure and opportunity to learn from others’ pupils across a lifetime – was not a consequence of technical or perceptual limitations associated with our stimuli.

While different limitations could in principal apply to *using* the signal as compared *to learning to use* it, the finding that observers could learn to use the same signal under conditions of increased "100% validity" again argues against the artificial nature of our paradigm obscuring any underling implicit capacity to *use* or *learn* the value of the pupil signal in more realistic, lower validity, conditions. This makes an interesting distinction between the apparent inability to exploit the pupil in a competitive situation to its apparent implicit use in emotion processing [5–8].

Because the “naïve-eye” condition always preceded the “informed-eye” condition, and we did see some evidence of learning in the 100% validity condition, one limitation of this design is that we cannot rule out a contribution of learning in the dramatic improvement of players in the “informed eye” condition. We consider this unlikely, however, as we would expect to see some traces of learning, such as a gradual increase in correct trials at the end of the “naïve eye” block. With the possible exception of one player, such an effect was, however, not observed in general (Figure 5a). Furthermore, most participants in the “reconstructed pupil”, who performed no other condition before, were also able to utilize the pupil dilations immediately (Figure 5c).

While we chose rock-paper-scissors for the present study because its intuitive rule structure allowed naïve participants to play without training, one strength of the game is that it shares some elements with more elaborate social exchanges and competitive situations. Pupil dilation is clearly sufficiently salient to be detected and could potentially be used in a range of social contexts. The question remains open, however, whether there exist any situations in which people are currently using pupil dilation or could be instructed on how to use the pupil to their advantage in more realistic scenarios.

In conclusion, our results show on the one hand that people could use pupil size as a cue in competitive interactions, but on the other hand render it unlikely that pupil dilation is being used in this way in everyday life. Although we cannot rule out that such situations exist, this seems in sharp contrast to emotional processing, where perceived pupil size modulates emotion perception and its neural substrate [5,8]. Given recent claims of "mind-reading" or "brain-reading" in the context of brain imaging (e.g., [1,2]), it remains remarkable a comparably simple physiological signal allows similar degrees of "mind-reading" in real-time. Even more remarkable, such "mind-reading" seems possible to nearly anyone using standard video equipment and the naked eye. This makes pupil dilation a signal utilizable for communication, which is of particular interest to patients with severe motor impairments [40].

## Supporting Information

Movie S1 **Example movie of an opponent’s pupil.**
Video depicting three games as the players viewed it in the informed-eye and naïve-eye conditions condition. If you want to try the experiment yourself, watch the movies and pick the best option to beat your opponent. The audio track consists of the words “rock”, “paper”, and “scissors”, presented in 4-s intervals in the identical (randomly ordered) sequence the opponent heard when the video was recorded. In case the video format does not work on your computer, various formats are available at http://www.staff.uni-marburg.de/~wetgast/rps/ (Correct answers for both movies: Opponent 1 selected “Rock” (3rd option). Opponent 2 selected “Paper” (1st option). Opponent 3 selected “Scissors” (2nd option). Winning options thus were “paper” in the first game, “Scissors” in the second and “rock” in the last).
(MOV)

Movie S2 **Example movie of an opponent’s reconstructed pupil.**
Video depicting the same three games shown in Movie S1 for the reconstructed-pupil condition.
(MOV)
